# Prevalence of Sarcopenic Obesity in Adults with Class II/III Obesity Using Different Diagnostic Criteria

**DOI:** 10.1155/2017/7307618

**Published:** 2017-03-22

**Authors:** Carlene A. Johnson Stoklossa, Arya M. Sharma, Mary Forhan, Mario Siervo, Raj S. Padwal, Carla M. Prado

**Affiliations:** ^1^Department of Agriculture, Food and Nutritional Science, University of Alberta, Edmonton, AB, Canada; ^2^Department of Medicine, University of Alberta, Edmonton, AB, Canada; ^3^Department of Occupational Therapy, University of Alberta, Edmonton, AB, Canada; ^4^Human Nutrition Research Centre, Institute of Cellular Medicine, Newcastle University, Newcastle on Tyne, UK; ^5^Department of Medicine, University of Alberta, Alberta Diabetes Institute, Edmonton, AB, Canada

## Abstract

*Background/Objective*. Sarcopenic obesity (SO) is a hidden condition of reduced lean soft tissue (LST) in context of excess adiposity. SO is most commonly reported in older adults and both its risk and prevalence increase with age. A variety of body composition indices and cut points have been used to define this condition, leading to conflicting prevalence and risk prediction. Here, we investigate variability in the prevalence of SO in an adult sample of individuals with class II/III obesity (BMI ≥ 35 kg/m^2^) using different diagnostic criteria.* Methods*. SO definitions were identified from a literature review of studies using dual-energy X-ray absorptiometry (DXA) to assess LST. Demographics, anthropometrics, and body composition (by DXA) were measured in *n* = 120, 86% female (46.9 ± 11.1 years).* Results*. LST was extremely variable in individuals, even with similar body sizes, and observed across the age spectrum. The prevalence of SO ranged from 0 to 84.5% in females and 0 to 100% in males, depending upon the definition applied, with higher prevalence among definitions accounting for measures of body size or fat mass.* Conclusion*. SO is present, yet variable, in adults with class II/III obesity. Accounting for body mass or fat mass may identify a higher number of individuals with SO, although risk prediction remains to be studied.

## 1. Introduction

Obesity is a complex chronic global disease affecting people worldwide across all ages, sexes, ethnicities, and nationalities [1]. Comparing body weight to height using body mass index (BMI) allows for the classification of obesity into class I (BMI 30–34.9 kg/m^2^), class II (BMI 35–39.9 kg/m^2^), and class III (BMI ≥ 40 kg/m^2^) [2] The prevalence of the highest obesity class is of concern due to its association with poorer health outcomes compared to lower BMI categories [1]. In 2013-2014, class III obesity affected 5.5% of males and 9.9% of females in the United States, with a linear increase in prevalence for females since 2005 [3]. Compared to the United States, fewer Canadians have class III obesity (2.5%); however the same trend by sex is found with a greater percentage of females affected (3%, compared to 2% of men) [4, 5].

In addition to BMI, waist circumference can be used to identify obesity. Both anthropometric methods provide a surrogate assessment of fat mass (FM) but are poor detectors of lean mass, also called lean soft tissue (LST), and hence of body composition. People with obesity can have varying proportions of LST and the single use of anthropometry to diagnose obesity precludes an accurate characterization of the different proportions of FM versus LST of an individual [6].

Emerging evidence suggests obesity can coexist with low LST (sarcopenia) [7–10]. In this case, the gravity impact of the excess body weight may not be sufficient to promote a concurrent increase in LST; therefore, individuals with obesity may have high FM without a parallel increase in LST [11]. Notably, this phenotype termed* sarcopenic obesity* may not be identified without the use of body composition assessment techniques. Low LST is an important prognostic factor in health and clinical conditions, as its main component is skeletal muscle mass, a tissue of vital importance for strength, functional mobility, immune function, and wound healing, among others [8, 12–16].

Sarcopenia has been primarily studied in older adults and individuals with chronic conditions but emerging evidence suggests that “healthy,” younger individuals are also at risk for presenting with this condition [17, 18]. Compounded with the consequences of excess FM, the concurrent sarcopenic obesity phenotype has been independently associated with worse morbidity and disability than either sarcopenia or obesity alone [10]. In the context of obesity treatment, weight loss results in the loss of both FM and LST [19, 20]. With repeated weight loss-gain cycles combined with age-related body composition changes, developing sarcopenic obesity is possible [10].

The identification of sarcopenic obesity is limited not only due to the availability of accurate body composition techniques but also due to heterogeneity in its diagnostic criteria [10]. A variety of body composition indices and cut-offs have been used to define sarcopenia and obesity, leading to conflicting findings on the prevalence and risk prediction of this condition [10, 21] Additionally, the great majority of studies have focused on identifying sarcopenic obesity in older adults and the prevalence within younger adults and those with class II/III obesity is not well defined. With the increasing prevalence of class III obesity [3] and of sarcopenia [18], the prevalence of sarcopenic obesity in these individuals is likely to increase dramatically. Therefore, the objective of this study is to explore the variability in the prevalence of sarcopenic obesity in an adult sample with class II/III obesity using different diagnostic criteria.

## 2. Methods

In a cross-sectional approach, we included consecutive patients from a multidisciplinary clinic providing medical and bariatric surgical interventions for adults (18–69 years) with class II/III obesity (BMI ≥ 35 kg/m^2^) in Edmonton, Alberta, Canada. Ethics approval was received from the University of Alberta Health Research Ethics Board.

Medical records were used to assess demographic and medical history obtained from the initial clinic assessment. Height was measured (without shoes, within 0.1 cm) with a wall-mounted stadiometer. Weight was measured (single layer of clothing, without shoes, within 0.1 kg) with a high-capacity weigh scale (Scale-Tronix 6702W®, WelchAllyn Inc., Skaneateles Falls, New York). Waist circumference was measured (within 0.1 cm) with a nonstretch tape at the midpoint of the torso (between lowest rib and iliac crest) on the right side using a cross-handed technique, recorded as the average of three consecutive measures.

Dual-energy X-ray absorptiometry (DXA) was required at the initial assessment and completed at a local medical imaging center [Hologic Discovery A (S/N 45026) or W (S/N 83792) scanners, software version 12.7.4.2, Hologic Inc., Bedford MA]. No subjects exceeded the DXA weight capacity limit (204 kg) or scan area length (195 cm). Reflection positioning was used for subjects with larger supine widths (>65 cm). Right side data was duplicated when values for the left side were either not reliable or available [22–24]. Collected values included whole body and segmental values for FM, LST, appendicular skeletal muscle mass (ASM, which is LST from arms and legs), and fat-free mass (FFM = LST + bone), and its derivatives are adjusted by height in square meters, also called indexes (e.g., FMI, ASMI). Detailed definitions of each of these body composition variables can be found elsewhere [6].

Subjects with complete initial clinic assessments and body composition analysis by DXA were included in the study. DXA scans available for analysis dated from January 2009 to June 2012, after which they were no longer ordered at the initial clinical assessment. All data was collected prior to starting obesity treatment. Subjects were excluded from the final analysis if DXA data was unreliable (i.e., segmental measurements were outside of the field of view or due to lack of separation of tissues between the arms and torso).

### 2.1. Sarcopenic Obesity: Definitions and Terminology

A literature search was conducted using PubMed, Scopus, and Web of Science databases to identify studies using definitions sarcopenic obesity based upon body composition data derived from DXA with or without use of anthropometric variables (e.g., weight, BMI, and waist circumference), excluding clinical studies (e.g., cancer). For definitions using ethnic-specific cut points, white/Caucasian references were included as the majority of our population (83.9% Edmonton, 86% Canada) self-identified as Caucasian [25]. Ethnicity was not collected as part of the clinic assessment, in accordance with the Freedom of Information and Protection of Privacy Act [26], therefore unavailable for analysis.

Based on the literature review, ten studies were identified using nine variables based upon LST or ASM to define sarcopenia (Table 1) and four variables were identified to define obesity (Table 2, plus FMI phenotype listed in Table 1) [7, 8, 18, 27–33].

The use of inconsistent body composition terminology may preclude a clear understanding of sarcopenic obesity's diagnostic criteria in the literature (i.e., authors use of different terminology for the same body composition variables). Therefore, in order to improve clarity while still accurately representing the body composition components being measured in each study, we consistently use the terms LST for studies measuring the nonbone, nonfat body compartment in general from the whole body (i.e., arms, legs, trunk, and head) and ASM for studies measuring LST from the arms and legs [6].

With the exception of BMI, each variable for sarcopenia and obesity used sex-specific cut points, with more than one cut point for some variables. Sixteen unique definitions (composed of a variable and cut point for each sarcopenia and obesity) were identified and applied to the sample to explore the prevalence of sarcopenic obesity. Linear regression analysis with ASM, height, and FM (kg) was used to determine prevalence of sarcopenia using the Newman et al., residual method [29]. The classification by body composition phenotypes was determined using deciles of population-derived ASMI and FMI cut points based on sex, BMI, and age, as per the protocol described in Prado et al. [18]. The classification of abnormal body composition phenotype as a load-capacity model (load being FM and capacity FFM) was calculated as the ratio of FM : FFM (as centiles), as per methodology described in Siervo et al. [33].

Additional classifications were derived from our study cohort, using ASMI calculated as the lowest 20th percentile and two standard deviations (SD) below the mean of the distribution; a method commonly reported in the literature when a reference population is not available [34]. Definitions of sarcopenic obesity utilizing measures of muscle strength or function were not included, as data were not available for our cohort.

### 2.2. Statistical Analysis

Descriptive statistics were used for subject characteristics, anthropometrics, and body composition and reported as mean (interquartile range). Normality testing was completed using the Shapiro-Wilk test. Frequencies and proportions were reported for categorical variables. Independent samples* t*-test for normally distributed data and nonparametric (Mann–Whitney* U*) independent samples* t*-test were used to compare variables between sexes. To account for missing data (waist circumference), subjects were compared to determine if differences existed between the groups. Correlations were tested using Pearson's* r* to explore the relationship between variables. Two-tailed tests were used for all the analysis with a *p* value of <0.05 considered for statistical significance. Data was analysed using IBM SPSS Statistics for Mac, version 23 (IBM Corp., Armonk, NY, USA).

## 3. Results

A total of 167 subjects with completed initial assessments and DXA scans were initially reviewed, in which 120 subjects (85.8% female) had reliable DXA data to be included in the final analysis. Clinical characteristics of excluded subjects (*n* = 47) were not different from those included in the analysis. Mean age of the entire cohort was 46.9 ± 11.1 years (range: 18–69 years). Subject characteristics, anthropometrics, and body composition are presented in Table 3.

Patients were community-dwelling (100%) and predominantly married/common-law (F 68%, M 65%) and worked outside the home (F 68%, M 70%) and 7.8% of females (no males) were current smokers. The majority of patients were generally well educated (F 98%, M 94% completed high school), with more females than males who completed their education at a university/college level (F 57%, M 35%). Recommended activity levels (>150 minutes of moderate intensity activities a week) were met by 20% of females and 23% of males.

**Figure 1:**
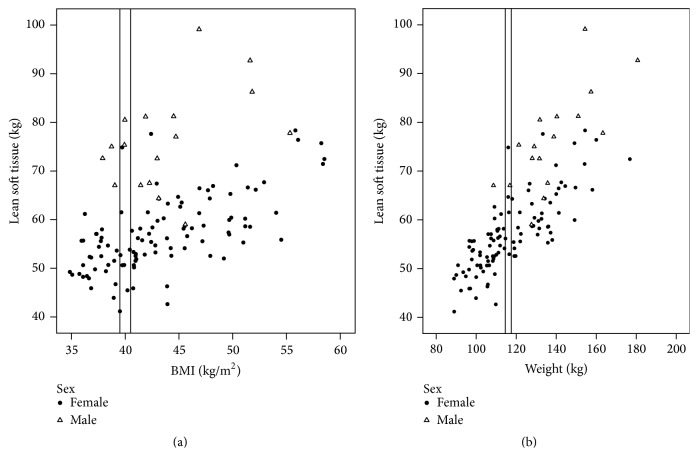
Variability of lean soft tissue by (a) body mass index (BMI) and (b) weight in adults with class II/III obesity (*n* = 120, females = 103). The box illustrates selected examples of females with (a) the same BMI (40 kg/m^2^) but LST varying from 41.2 to 74.9 kg and (b) same weight (116 kg) but LST varying from 52.9 to 74.9 kg.

Due to the positively skewed data for weight in females, some variables were not normally distributed. Independent samples* t*-tests and nonparametric (Mann–Whitney* U*) tests results were compared and showed the same results. No significant differences were observed between females and males for age, BMI, and FM (kg), Table 3. Compared to males, females had higher values for FM (%), FMI, and FM : FFM ratio and lower values for variables depicting the lean mass compartment. A large variability in LST (kg) was observed for individuals with the same body size, Figures 1(a) and 1(b). The relationship between BMI and LST in females and males was moderate and weak (*R* = 0.41,* R* = 0.20, resp.), Figure 1(a).

The entire cohort met the criteria for obesity defined by BMI, waist circumference, and FMI cut points (Table 2). For FM%, all males exceeded the five different cut points. One female (BMI 39.7 kg/m^2^ and 32.2% FM) did not meet the criteria for obesity defined by % FM with five of the six different cut points. Ten females (9.7%) had % FM below the highest cut point (42.9%) and therefore would not be identified with obesity despite BMI's ranging from 35.9 to 45.1 kg/m^2^. Of note, the highest sex-specific 20th percentile for FMI was >23.8 kg/m^2^ for females and >21.5 kg/m^2^ for males.

Considering the entire cohort had class II/III obesity as defined by BMI, when each definition of sarcopenia was applied to the current sample, the prevalence of sarcopenic obesity varied from 0 to 84.5% for females and 0 to 100% for males (Table 4). Definitions using unadjusted values for LST, ASM, or ASMI, with the exception of the highest ASMI cut point, failed to identify any subjects with sarcopenic obesity. Notably, a higher prevalence of sarcopenic obesity was identified by definitions combining ASM either with weight, BMI, or a measure of FM.

The sex-specific cut points developed from the Newman et al. [29] study group were only able to identify males with sarcopenic obesity in our cohort, Table 4. Applying the Newman et al. [29] residual method to derive cut points from the current cohort, sarcopenic obesity was identified in both sexes. For the latter, the cohort-specific cut points derived from the 20th percentile of the sex-specific distributions of the residuals were <2.96 for females and <−4.82 for males, identifying 19.4% of females and 17.6% of males with sarcopenic obesity.

**Figure 2:**
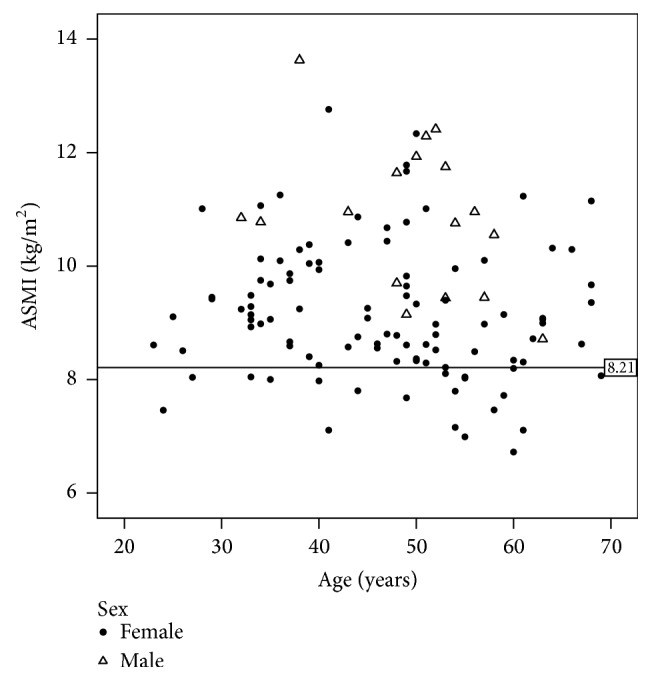
Variability of appendicular skeletal mass index (ASMI) by age (23–69 years) in adults with class II/III obesity (*n* = 120, females = 103). The line indicated the 20th percentile of ASMI for females; subjects below this level ranged in age from 24 to 69 years.

Equivalent cut points for ASMI were also derived from the study cohort. The cohort-specific 20th percentile cut point to describe sarcopenic obesity by ASMI was <8.21 kg/m^2^ for females and <9.44 kg/m^2^ for males. Using the lowest 2 SD criteria for ASMI, the cohort-specific cut points were <6.79 kg/m^2^ for females and <8.62 kg/m^2^ for males. Selecting the cohort-specific 20th percentile, low ASMI was observed across the age spectrum, Figure 2.

**Figure 3:**
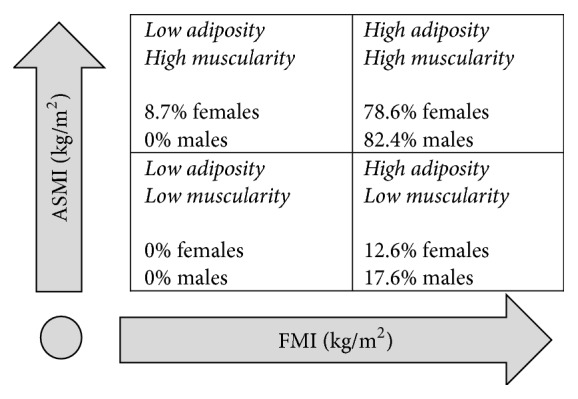
Body composition phenotype, by decile groups of appendicular skeletal mass index (ASMI) and fat mass index (FMI), for adults with class II/III obesity (*n* = 120, females = 103) [18].

Using the phenotype definition proposed by Prado et al. [18] to the entire sample, 16 (13.3%) subjects where classified with high adiposity and low muscularity (sarcopenic obese-like phenotype), and 95 (79.2%) subjects presented with the high adiposity and high muscularity phenotype (obese nonsarcopenic-like phenotype), Figure 3. Nine females were classified as having a normal body composition phenotype.

Using the load-capacity model to account for the interaction of both body compartments [33], the FM : FFM ratio identified about a third of females and three-quarters of males with moderate and severe body composition phenotype (≥85th percentile), Table 4.

## 4. Discussion

Previous research identified sarcopenic obesity in older adults [35] and groups with certain chronic diseases [6]. Although several diagnostic criteria have been used, no one approach has been widely accepted. This is the first study to use state-of-the-art methodology (DXA) to explore the prevalence of sarcopenia in a younger adult cohort (mean age 46.9 ± 11.1 years) with class II/III obesity. LST was extremely variable in individuals with similar BMI illustrating a wide variability of body composition within the same body size. Using 16 previously reported definitions, the prevalence of sarcopenic obesity varied from zero to 100%. Such variability precludes a comprehensive understanding of the prevalence of sarcopenia in younger individuals with more severe classes of obesity, as well as the development of preventive and treatment strategies for this condition in clinical settings. As these individuals are actively seeking obesity treatment, maintaining lean mass should be a coprimary endpoint of the nutrition care plan together with weight management.

All males and almost all females were classified with obesity using diverse FM% cut points (30–42.9% for females and 20–29% for males). Most individuals with a BMI ≥ 35 kg/m^2^, excluding extremely muscular individuals, will present with excess adiposity [36] and prevalence will vary only based on the comparison cohort used to identify the cut point. For example, 10 females from our cohort would not be considered to have obesity using the Zoico et al. [27] cut point based on quintiles of % FM from a sample of healthy elderly females (BMI 26 ± 3.8 kg/m^2^). Nonetheless, these 10 females were within 0.2% to 3.7% below the % FM cut point. Interestingly, using the adjusted Prado et al. cut points [18], we observed that nine females were not classified as having high adiposity. In addition to sex, this cut point is notably adjusted for age and BMI. Six females were identified as having both lower % FM and FMI using the Zoico et al. [27] and Prado et al. [18] cut points respectively.

Prevalence of sarcopenia ranged approximately from 0 to 84.5% in females and from 0 to 100% in males. The null prevalence using several cut points may be explained by the approach used to define sarcopenia. Reference values to diagnose sarcopenia have been primarily developed from older cohorts, which may not be applicable for younger adults. Although Cherin et al. [17] included younger individuals (45–83 years), their cohort's mean age was 63.1 ± 10.2 years and the prevalence of sarcopenic obesity was not reported.

In our study, no subjects were identified with sarcopenic obesity by definitions of LST [27, 28]. Baumgartner et al. [7, 37] and others have defined sarcopenia using ASMI sex-specific cut points based on two standard deviations below the mean for a young reference group [7, 21, 30, 37–39]. No subjects were identified with sarcopenic obesity applying each of these different cut points. Although these young reference groups were North American and of similar age to the current study cohort, their BMI (described as “normal”) would be much lower. However, sarcopenic obesity may still be present but not identified as the cut points may not be sensitive enough to identify relatively low lean mass in subjects with larger total body mass. Likewise, no subjects were identified as sarcopenic using Newman et al. cut point which defined sarcopenia as the lowest 20th percentile of their cohort's ASMI distribution [29]. Notably, applying the same method to our cohort, our ASMI cut points were 45% and 31% greater for females and males, respectively, highlighting how differences in age and body size may impact comparison among different cohorts.

Although the quantity of LST may meet or exceed reference values derived from normal, healthy reference populations (e.g., normal BMI or age 25 years), the higher LST amount is insufficient to maintain the larger body size (largely due to a larger FM amount). This phenomenon can be conceptualized as the metabolic load (due to FM) versus capacity (of the LST/FFM) model previously described [33]. Therefore, sarcopenia in those with obesity may be present at higher LST values and must be evaluated in relation to body mass or FM.

Our findings support the use of a combined definition of body mass or FM to a measure of sarcopenia for the identification of sarcopenic obesity in this cohort of younger adults with class II/III obesity. Considering measures of ASM with weight [31, 32], BMI [8], FMI [18], or FM [29, 33], our prevalence of sarcopenia ranged from 12.6 to 84.5% for females and 17.6 to 100% for males. Likewise, in the Newman et al. [29] study, higher prevalence rates were observed for both sexes using a method in which ASM was considered in relation to height and FM compared to none using nonadjusted ASMI cut points. The authors concluded this technique captured the effect of both LST (as ASM) and high FM simultaneously, therefore identifying a greater proportion of people with obesity as being sarcopenic. Our findings are consistent with their results and highlight the potential importance of considering FM with LST indices together when evaluating sarcopenia in people with obesity.

We were able to identify three body composition phenotypes using the Prado et al. [18] previously established cut points, where age, sex, and BMI-specific reference curves were created to define body composition phenotypes (FMI and ASMI above or below the 50th percentile). As the 50th percentile was used, the terms “obesity” and “sarcopenia” were avoided with individuals being classified using a combination of high/low adiposity and high/low muscularity. The concurrent high adiposity and low muscularity are the “sarcopenic obesity-like” phenotype with an observed population prevalence of 10.3% in females and 15.2% in males. Although subjects >136 kg were excluded from that study thereby limiting the reference data, applying this method to the current study cohort produced similar results, identifying 12.6% of females and 17.6% of males with sarcopenic obesity. Similar to Siervo et al. [33] FM : FFM ratio reference curves in our study found females had a higher FM : FFM ratio than males. Notably, the current study included subjects with higher weights, with 17.5% of females and 41.2% of males >136 kg. The load-capacity model is a novel method to identify low LST relative together with excess FM in subjects with class II/III obesity.

Although our sample size of males was small, their prevalence of sarcopenia was higher than females for all definitions except the Newman et al. residual method [29], where the prevalence was similar. The prevalence of sarcopenia by sex is controversial with some studies reporting higher prevalence among males, others among females and some finding no differences [35].

An important consideration for any definition is to understand the characteristics of the group from which the cut points were derived. Notably, some definitions were developed from European or Asian cohorts that are ethnically different from a North American population. Widely recognized differences in body composition among different ethnicities preclude a direct comparison of sarcopenic obesity prevalence among different studies.

In the absence of a young reference group, cohort-specific cut points were used using the lowest one [30] or two [28, 29] quintiles for ASMI. Applying this approach to our dataset, our cohort-specific cut points were much higher than previously published ones, again highlighting that cut points derived from other cohorts or nonspecific populations (i.e., older adults, individuals without obesity) may either fail to detect or underestimate the prevalence of sarcopenic obesity in adults with class II/III obesity. Contrary to expectations, the prevalence of sarcopenia was not higher among individuals ≥65 years compared to those <65 years [18]. Indeed, we reported ASMI was highly variable across the age spectrum; only one of the 23 individuals with an ASMI below the 20th percentile for this cohort was older than 65 years (Figure 2).

The large variability of LST (Figures 1(a) and 1(b)) in individuals with the same body size represents a clinical challenge for determining nutritional requirements. For example, protein and energy needs are often determined based on body weight, yet, considering lean mass drives protein requirements, people with the same body weight can receive varying amounts of protein per unit of lean mass (LST), a concept previously explored [40, 41]. In the selected example on Figure 1(b), if protein requirements were assessed as 1 g/kg actual body weight (116 kg), the estimated amount of dietary protein would be equivalent to 1.6 to 2.2 g/kg LST.

Data on body composition of adults with class II/III obesity is limited, especially of those with BMI > 40 kg/m^2^. One barrier is related to equipment limitations [42]. Individuals with class III obesity not only have increased weights, but increased body dimensions such as height or supine width. Although there are large body composition data sets available, subjects above 136 kg were excluded due to equipment limitations [36]. Recent DXA equipment improvements, such as the Lunar iDXA (GE Healthcare) and Discovery/Horizon models (Hologic, Inc.), have increased scan area widths and weight capacities, improving the capability to assess more people with obesity.

Notably, this study was completed prior to initiation of obesity treatment at the clinic. Weight loss is associated with reductions in both FM and LST, with weight regain predominately as FM [10]. If people with low LST are not identified as such, initiating obesity treatments targeted to reduce weight can further reduce LST, thereby either creating or worsening a sarcopenic state.

Limitations of our study include our ambulatory cohort seeking obesity treatment, which may not reflect all adults with obesity or other care settings (i.e., acute care, long term care). Although the representation of males in the current study appears low, it is comparable to other studies conducted in this clinic [43, 44]. In general, males tend to be underrepresented in obesity treatment studies [45, 46]. Additionally, we were unable to explore definitions of sarcopenia using a measure of muscle function, as these were not collected as part of patient's initial assessment.

## 5. Conclusions

Sarcopenia was present in our cohort but masked by obesity. Basic anthropometric measurements alone are inadequate to identify sarcopenia and hence sarcopenic obesity in these individuals. Sophisticated tools such as DXA may be needed to identify and profile LST of adults with class II/II obesity and could be implemented as part of clinical assessment. The inclusion of measures of FM and body size in the definition of sarcopenic obesity identifies a greater proportion of individuals with this abnormal body composition phenotype compared to stand-alone definition of low lean mass. Different diagnostic criteria should be tested in prospective studies investigating the risk prediction for metabolic, functional, and clinical parameters of these adults with class II/III obesity.

## Figures and Tables

**Table 1:** Variables and methods used to define sarcopenia amongst studies investigating sarcopenic obesity using dual-energy X-ray absorptiometry.

Variables^a^	Reference	Study group	Method for sex-specific cut points	Females	Males
LSTI (kg/m^2^)	Zoico et al., 2004 [27]	Older females (67–78 y) Italy	Lowest 2 quintiles of the distribution of young reference group (female, 20–50 y).	<5.7	NA
LST by weight × 100 (%)	Zoico et al., 2004 [27]	Older females (67–78 y) Italy	Class I: 1 SD below mean, Class II: 2 SD below mean of young reference group (female, 20–50 y).	23.1–26.7 <23.1	NA
	Kim et al., 2009 [28]	Adults (20–88 y) Korea	2 SD below mean of young reference group derived from study group.	<30.7	<35.71
ASM (kg)	Batsis et al., 2015 [8]	Older adults (>60 y) United States	Classification and regression tree analysis of older adults (>65 y).	<15.02	<19.75
ASMI (kg/m^2^)	Zoico et al., 2004 [27]	Older females (67–78 y) Italy	Class I: 1 SD below mean, Class II: 2 SD below mean of young reference group (female, 20–50 y).	4.7–5.6 <4.7	NA
	Kim et al., 2009 [28]	Adults (20–88 y) Korea	2 SD below mean of young reference group (20–40 y).	<5.14	<7.40
	Baumgartner et al., 1998 [7]	Older adults (>64 y) United States	2 SD below mean of young reference group (18–40 y).	<5.45	<7.26
	Newman et al., 2003 [29]	Older adults (70–79 y) United States	Lowest quintile (20th percentile) of study group.	<5.67	<7.23
	Bouchard et al., 2009 [30]	Older adults (68–82 y) Canada	2 SD below mean of young reference group (20–35 y).	<6.29	<8.51
	Kim et al., 2009 [28]	Adults (20–88 y) Korea	Lowest two quintiles (40th percentile) of study group.	<7.36	<8.81
ASM by weight × 100 (%)	Levine and Crimmins, 2012 [31]	Older adults (>60 y) United States	2 SD below mean of young reference group (20–40 y).	<19.43	<25.72
	Oh et al., 2015 [32]	Older adults (>60 y) Korea	1 SD below mean of young reference group (20–39 y).	<23.4^b^	<29.6^b^
ASM by BMI (kg/m^2^)	Batsis et al., 2015 [8]	Older adults (>60 y) United States	Classification and regression tree analysis of older adults (>65 y).	<0.512	<0.789
ASM by height, FM (residuals)	Newman et al., 2003 [29]	Older adults (70–79 y) United States	Lowest quintile (20th percentile) of the distribution of residuals of study group.	<−1.73	<−2.29
ASMI and FMI (phenotype)	Prado et al., 2014 [18]	Adults (>18 y) United States	Age, sex, and BMI-specific reference curves, by decile (>18 y).	HA-LM^c^	HA-LM^c^
FM : FFM ratio	Siervo et al., 2015 [33]	Adults (>18 y) United States	Age-standardized reference curves, stratified by sex and BMI, by centile (>18 y).	≥85th percentile	≥85th percentile

^a^Terminology for variables selected for consistency and may differ from terms used by original authors; these depict the correct compartment being measured. ^b^Cut  points determined from reported sex-specific mean and standard deviation in Oh et al., 2015 [32]. ^c^HA-LM: high adiposity (FMI 50–100) and low muscle mass (ASMI 0–49.99) with individual *z*- scores based upon age, sex, and BMI. LSTI: lean soft tissue index; y: years; NA: not applicable; LST: lean soft tissue; SD: standard deviation; ASM: appendicular skeletal muscle mass; ASMI: appendicular skeletal mass index; BMI: body mass index; FM: fat mass; FMI: fat mass index; FFM: fat-free mass.

**Table 2:** Prevalence of obesity in study cohort (*n* = 120) using various sex-specific definitions determined by anthropometric and dual-energy X-ray absorptiometry measurements amongst studies investigating sarcopenic obesity.

Variables	Reference	Females (*n* = 103)		Males (*n* = 17)	
		Cut point	Prevalence (%)	Cut point	Prevalence (%)
BMI (kg/m^2^)	Newman et al., 2003 [29]	≥30	100	≥30	100
	Oh et al., 2015 [32]				
Waist circumference (cm)^a^	Levine and Crimmins, 2012 [31]	>88	100	>102	100
Fat mass (%)	Kim et al., 2009 [28]	>31.71	100	>20.21	100
	Bouchard et al., 2009 [30]	≥35	99	≥28	100
	Batsis et al., 2015 [8]	≥35	99	≥25	100
	Baumgartner et al., 1998 [7]	>38	99	>27	100
	Baumgartner et al., 2004 [37]	>40	98	>28	100
	Zoico et al., 2004 [27]	>42.9	90.3	NA	

^a^Waist circumference not available for the entire cohort: females (*n* = 81, 78.6%) and males (*n* = 13, 76.5%). BMI: body mass index; NA: not applicable.

**Table 3:** Subject characteristics, anthropometrics, and body composition (*n* = 120), by sex.

Variables^a^	Females (*n* = 103)	Males (*n* = 17)	*p* value
	Mean (IQR)	Mean (IQR)	
Age (years)	46.5 (18)	49.4 (10)	0.352

*Anthropometrics*			

Height (cm)	164.1 (8.3)	177.2 (9.7)	<0.0001
Weight (kg)	117.3 (25.8)^b^	138.2 (24.9)	<0.0001
BMI (kg/m^2^)	43.5 (8.4)^b^	44.0 (6.3)	0.960
Waist (cm)^c^	120.4 (18.2)	141.0 (15.3)	<0.0001

*Body composition*			

Fat mass (kg)	55.6 (16.8)^b^	56.5 (17.5)	0.759
Fat mass (%)	48.0 (5.7)	41.4 (8.3)	<0.0001
FMI (kg/m^2^)	20.6 (5.1)^b^	18.0 (6.0)	0.009
FM : FFM ratio	0.9 (0.2)	0.7 (0.2)	<0.0001
LST (kg)	57.1 (9.3)^b^	76.2 (13.9)	<0.0001
LSTI (kg/m^2^)	21.2 (2.6)^b^	24.2 (3.9)	<0.0001
LST by weight × 100 (%)	49.0 (6.4)	55.4 (9.1)	<0.0001
ASM (kg)	24.7 (4.8)^b^	34.2 (7.3)	<0.0001
ASMI (kg/m^2^)	9.2 (1.6)	10.9 (2.3)	<0.0001
ASM by weight × 100 (%)	21.2 (2.9)	24.9 (5.3)	<0.0001
ASM by BMI	0.57 (0.1)^b^	0.78 (0.2)	<0.0001

^a^Terminology for variables is selected for consistency and may differ from terms used by original authors. ^b^Variable not normally distributed. ^c^Waist circumference not available for the entire cohort: females (*n* = 81, 78.6%) and males (*n* = 13, 76.5%). IQR: interquartile range; BMI: body mass index; FMI: fat mass index; FM: fat mass; FFM: fat-free mass; LST: lean soft tissue; LSTI: lean soft tissue index; ASM: appendicular skeletal mass; ASMI: appendicular skeletal mass index.

**Table 4:** Prevalence of sarcopenic obesity in the study cohort (*n* = 120) using various sex-specific definitions determined by anthropometric and dual-energy X-ray absorptiometry measurements amongst studies investigating sarcopenic obesity.

Variables^a^	Reference	Females (*n* = 103)		Males (*n* = 17)	
		Cut point	Prevalence (%)	Cut point	Prevalence (%)
LSTI (kg/m^2^)	Zoico et al., 2004 [27]	<5.70	0	NA	NA
LST by weight × 100 (%)	Kim et al., 2009 [28]	<30.70	0	<35.71	0
	Zoico et al., 2004 [27]	(I) 23.1–26.7 (II) <23.1	0 0	NA NA	NA NA
ASM (kg)	Batsis et al., 2015 [8]	<15.02	0	<19.75	0
ASMI (kg/m^2^)^b^	Zoico et al., 2004 [27]	(I) 4.7–5.6 (II) <4.7	0 0	NA NA	NA NA
	Kim et al., 2009 [28]	<5.14	0	<7.40	0
	Baumgartner et al., 1998 [7], 2004 [37]	<5.45	0	<7.26	0
	Newman et al., 2003 [29]	<5.67	0	<7.23	0
	Bouchard et al., 2009 [30]	<6.29	0	<8.51	0
	Kim et al., 2009 [28]	<7.36	4.9	<8.81	5.9
ASM by weight × 100 (%)	Levine & Crimmins, 2012 [31]	<19.43	23.3	<25.72	58.8
	Oh et al., 2015^c^ [32]	<23.4	84.5	<29.6	100
ASM by BMI (kg/m^2^)	Batsis et al., 2015 [8]	<0.512	18.4	<0.789	47.1
ASM adjusted for height and fat mass (residuals)^b^	Newman et al., 2003 [29]	<−1.73	0	<−2.29	23.5
ASMI and FMI (phenotype)	Prado et al., 2014 [18]	HA-LM^d^	12.6	HA-LM^d^	17.6
FM : FFM ratio	Siervo et al., 2015 [33]	≥85th percentile	28.2	≥85th percentile	76.5

^a^Terminology for variables is selected for consistency and may differ from terms used by original authors. ^b^Where applicable, equivalent cut points derived from the study-specific cohort are listed in the text. ^c^Cut points determined from reported sex-specific mean and standard deviation in Oh et al., 2015[32]. ^d^HA-LM: high adiposity (FMI 50–100) and low muscle mass (ASMI 0–49.99) with individual *z*-scores based upon age, sex, and BMI. LSTI: lean soft tissue index; NA: not applicable; LST: lean soft tissue; ASM: appendicular skeletal muscle mass; BMI: body mass index; ASMI: appendicular skeletal mass index; FMI: fat mass index; FM: fat mass; FFM: fat-free mass.
